# Supplementary material for: Effective Therapy Using a Liposomal siRNA that Targets the Tumor Vasculature in a Model Murine Breast Cancer with Lung Metastasis
Source: Mol Ther Oncolytics. 2018 Oct 30;11:102–8. doi: 10.1016/j.omto.2018.10.004 (PMC6280606; doi:10.1016/j.omto.2018.10.004)
Supplement: Document S1. Figures S1–S8 [file mmc1.pdf]

OMTO, Volume *11*

Supplemental Information

Effective Therapy Using a Liposomal siRNA that Targets the Tumor Vasculature in a Model Murine Breast Cancer with Lung Metastasis

Yu Sakurai, Tomoya Hada, Akari Kato, Yuta Hagino, Wataru Mizumura, and Hideyoshi Harashima

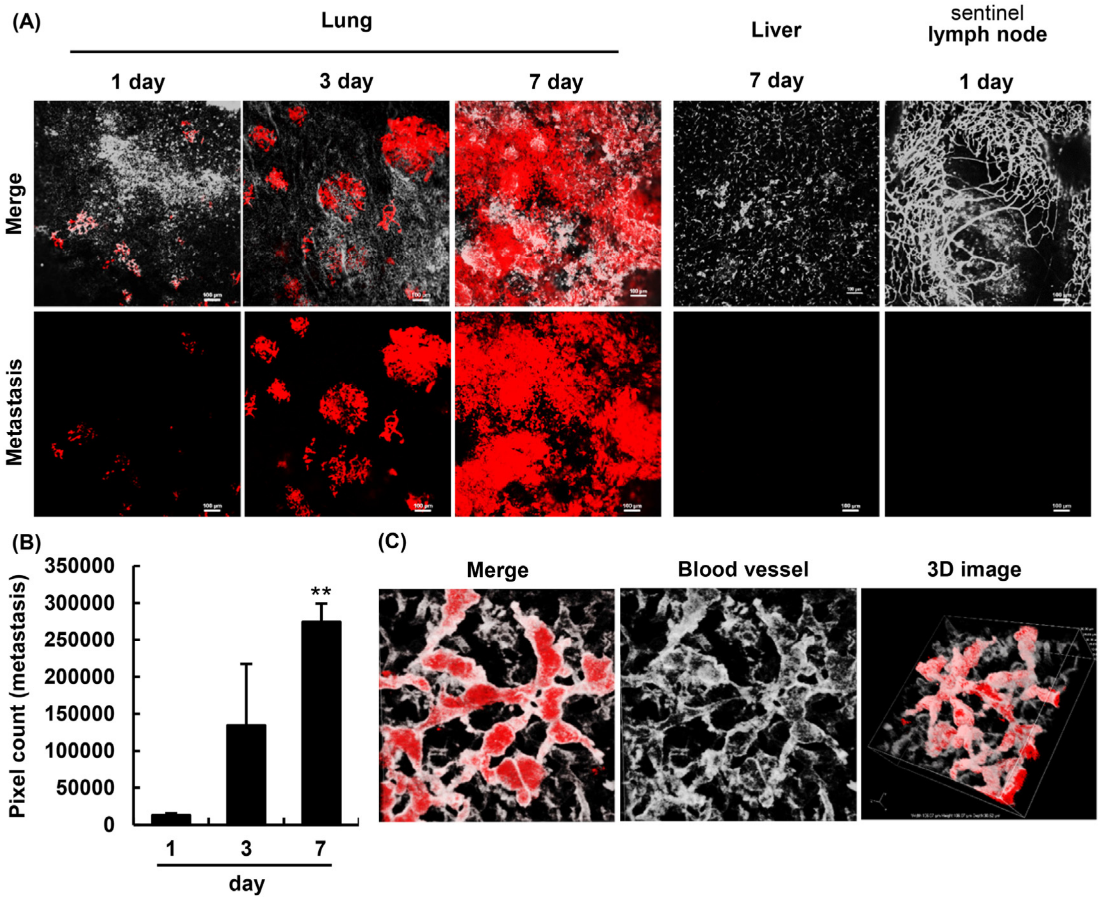

Supplemental Figure 1 Proliferation properties of 4T1-tdTomato/luc cells in the lung. (A) Confocal images of metastasized lung, liver and sentinel lymph node were observed at 1, 3 or 7 days after the inoculation. Red and gray dots indicate 4T1 cells and vessels, respectively. Bars are 100 μm. (B) Red pixels were counted by ImageJ software. Statistical analysis was performed by ANOVA, followed by SNK test. (n=9). **: $p < 0.01$. (C) 3D images of the lung were observed 1 day after the inoculation.

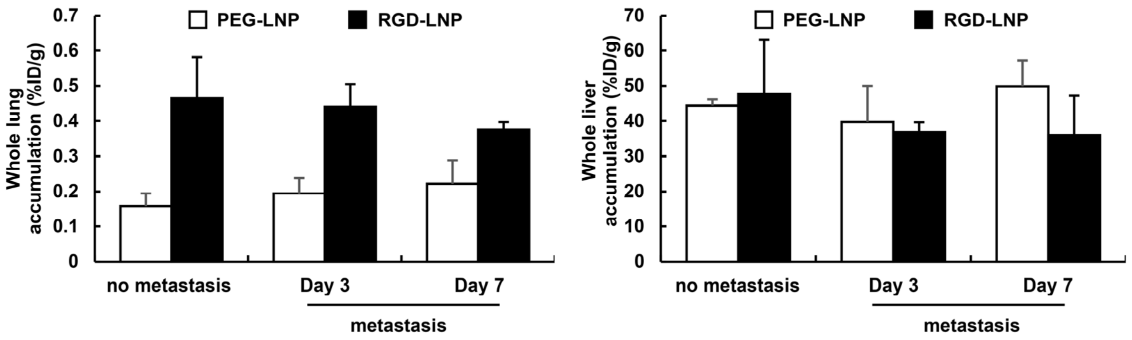

Supplemental Figure 2 PEG- and RGD-LNP accumulation in the lung and liver. Normal mice or metastasized mice were inoculated with radioisotope-labeled LNPs at 3 days or at 7 days. Lung and liver were excised 24 hours after the injection of LNPs, and the radio-activity in the dissolved lung and liver was detected by liquid scintillation counting. Data represent the mean ± SD.

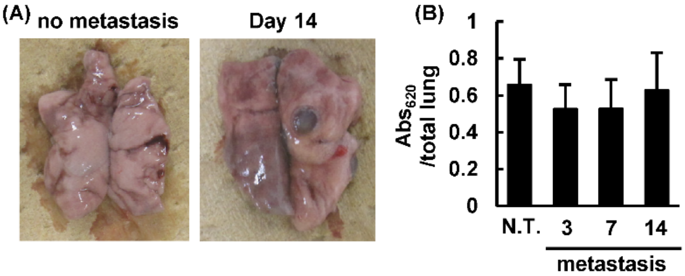

Supplemental Figure 3 Permeability of the metastasized lung vasculature. (A) Visual images of lung samples from mice that were treated with Evans Blue dye. (B) The absorbance of evans blue dye was measured by absorption spectrophotometer at 605 nm after the excised lungs were exhaustively minced. n=3~6. Data represent the mean ± SD.

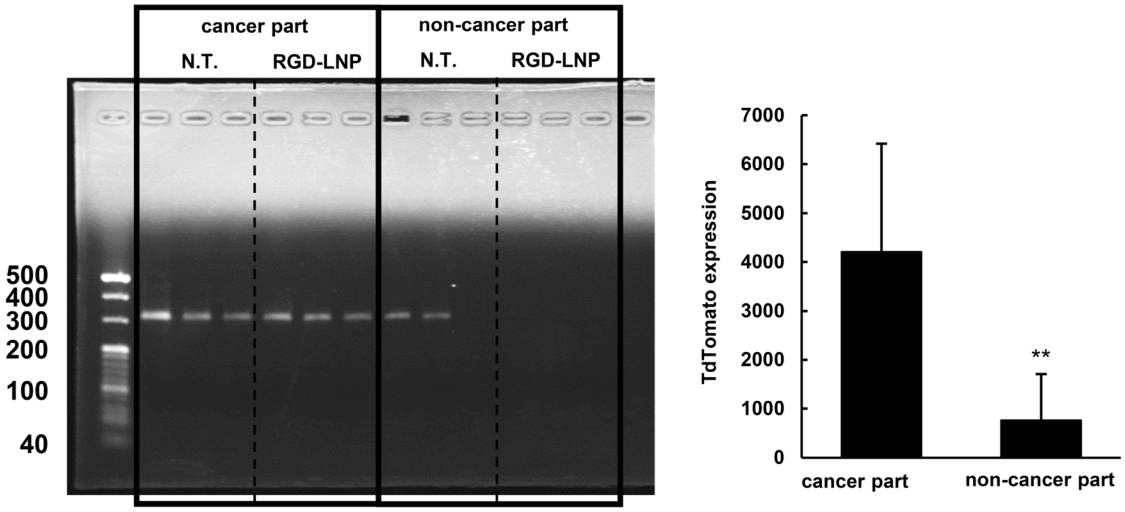

Supplemental Figure 4 Confirmation that the cancerous region was isolated from the non-cancerous region. Cancer and non-cancer regions were isolated based on visual observation, and total RNA was then extracted with TRIzol (Invitrogen). The tdTomato mRNA was then amplified by nested PCR, and the PCR amplicons were then detected by agarose electrophoresis. The electrophoresis images were quantified by ImageJ software. Statistical analyses were performed using the Student's t-test. **: $p < 0.01$ (n=6). Data represent the mean ± SD.

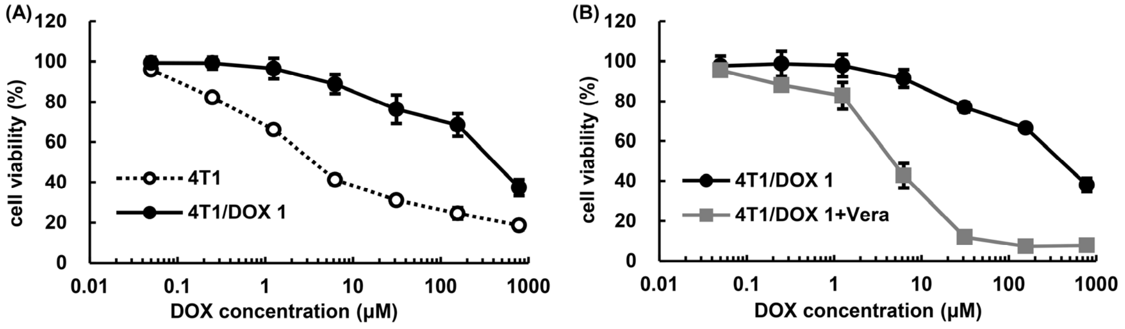

Supplemental Figure 5 Characterization of doxorubicin (DOX)-resistant 4T1 cells (4T1/DOX 1). (A) The sensitivity of 4T1 and 4T1/DOX 1 cells against DOX was measured after treating the cells with 0 – 780 μM DOX. The viability of cells was determined by a WST-8 assay. (B) To elucidate the mechanism for DOX resistance, cells were treated with 0.1 mM Verapamil 1 hr before the addition of DOX (Vera). Data represent the mean ± SD.

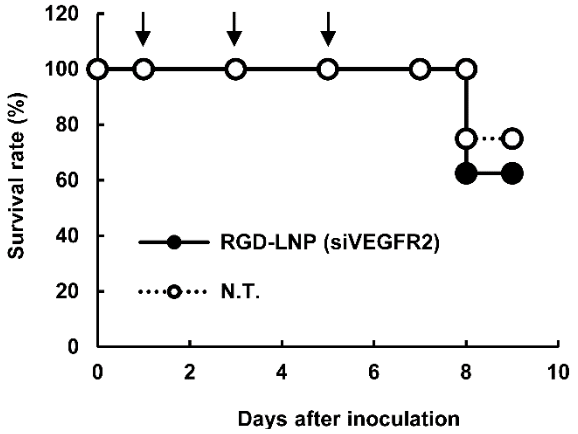

Supplemental Figure 6 The therapeutic effect of RGD-LNP encapsulating siRNA against VEGFR2 (siVEGFR2). RGD-LNP containing siVEGFR2 was administered via the tail vein at a dose of 3.0 mg/kg. Arrows denote the points of injection.

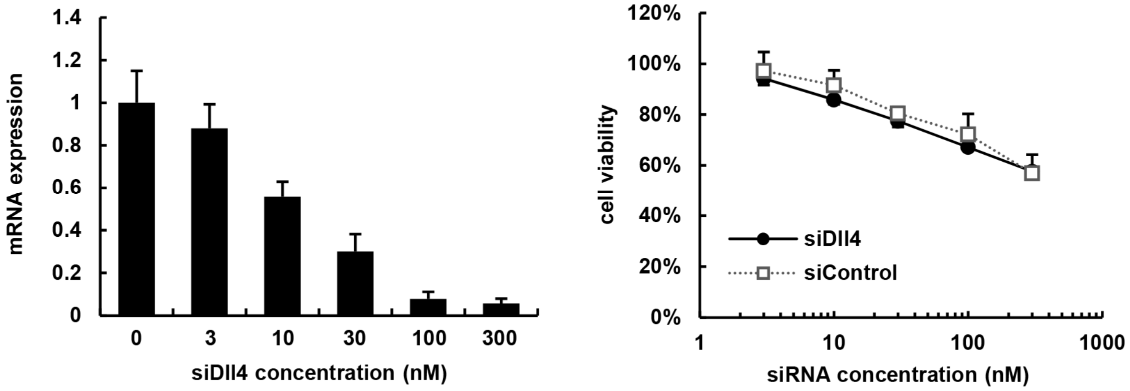

Supplemental Figure 7 Cell killing effect by siDll4. Cells were transfected with siDll4 or siRNA against human polo-like kinase 1 (siControl) by RNAiMAX at the indicated concentrations. Dll4 expression was determined by quantitative RT-PCR. The viability of cells was determined by a WST-8 assay 24 hours after the transfection. Data represent the mean ± SD.

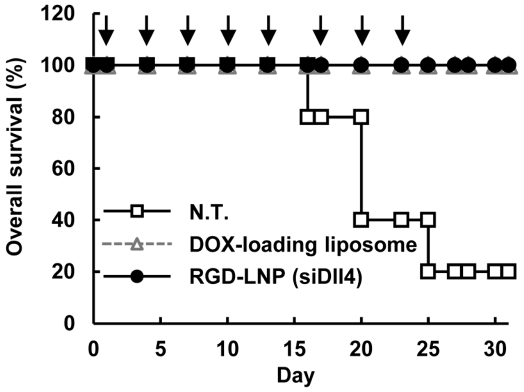

Supplemental Figure 8 The therapeutic effect of RGD-LNP and DOX-loaded liposome in DOX-sensitive 4T1 cells. Mice were treated with RGD-LNP encapsulating siDll4 or DOX-loaded liposome at 3 mg siRNA/kg or 3 mg DOX/kg. Arrows indicate the point of injection of the therapeutics.
