# Supplementary material for: Effective Therapy Using a Liposomal siRNA that Targets the Tumor Vasculature in a Model Murine Breast Cancer with Lung Metastasis
Source: Mol Ther Oncolytics. 2018 Oct 30;11:102–8. doi: 10.1016/j.omto.2018.10.004 (PMC6280606; doi:10.1016/j.omto.2018.10.004)
Supplement: Document S2. Article plus Supplemental Information [file mmc2.pdf]

Molecular Therapy
Oncolytics
Original Article

Effective Therapy Using a Liposomal siRNA that Targets the Tumor Vasculature in a Model Murine Breast Cancer with Lung Metastasis

Yu Sakurai,[1] Tomoya Hada,[1] Akari Kato,[1] Yuta Hagino,[1] Wataru Mizumura,[1] and Hideyoshi Harashima[1]

[1]Molecular Design of Pharmaceutics, Faculty of Pharmaceutical Sciences, Hokkaido University, Kita 12, Nishi 6, Kita-ku, Sapporo 060-0812, Japan

Although metastatic cancer is a major cause of death for cancer patients, no efficacious treatment for metastasis is available. We previously showed that the growth of a primary tumor could be inhibited by the administration of an anti-angiogenic small interfering RNA (siRNA) that is encapsulated in an RGD peptide-modified lipid nanoparticle (RGD-LNP). We herein report on the delivery of siRNA by an RGD-LNP to the vasculature is also effective for treating metastatic tumors. We compared the RGD-LNP with the polyethylene glycol (PEG)ylated LNP (PEG-LNP) in terms of accumulation in a lung-metastasized model. Despite malformed structure of vasculature in the metastasized lung, the accumulation of the PEG-LNP in the metastasized lung was lower than that for the RGD-LNP, which suggests that the delivery strategy based on vascular permeability is not completely effective for targeting metastasis tumors. The systemic injection of the RGD-LNP induced a significant silencing in the metastasized vasculature, but not in the normal lung. In addition, the continuous injection of the RGD-LNP encapsulating siRNA against a delta-like ligand 4 (DLL4) drastically prolonged the overall survival of metastasized model mice. Accordingly, our current findings suggest that vasculature targeting would be more effective than enhanced permeability and retention effect-based therapy for the treatment of metastatic cancer.

INTRODUCTION

Metastasis is a major cause of cancer-related death.[1,2] The 5-year survival rate has not been improved in the period from 2005 to 2015, despite the tremendous advances in cancer treatment, while that for cancer patients without distant metastasis has been improved.[3] Thus, an innovative therapy is needed for the treatment of advanced, metastatic cancer.

According to the previous report showing that tumor endothelial cells (TECs) in lymphatic metastasis tumors comprised a supportive phenotype as well as in primary tumors,[4] we focused on the use of TECs as a target for the treatment of metastatic tumors. To regulate a gene of interest, we previously developed a TEC-targeting small interfering RNA (siRNA)-loaded lipid nanoparticle (LNP) (RGD-LNP), which was composed of a fusogenic cationic lipid (YSK05) and a cyclic RGD peptide that recognized $\alpha V\beta 3$ integrin, which is highly expressed in TECs.[5,6] Systemically injected RGD-LNP caused the efficient knockdown in TECs, with an ED_{50} of approximately 0.75 mg/kg in a human renal cell carcinoma model.[7] Angiogenesis-related gene vascular endothelial growth factor receptor 2 (VEGFR2), which is inhibited by RGD-LNP, resulted in a delay in the growth of the primary tumor.

In previous studies, cationic nanoparticles have frequently been used to target the vasculature of a metastasis site, because cationic liposomes were reported to readily accumulate in angiogenic vessels in tumors and sites of chronic inflammation.[8] For example, Santel et al.[9] reported that siRNA lipoplex AtuPlex, which contained the cationic lipid AtuFect, inhibited tumor growth in a pulmonary metastasis model. Dahlman et al.[10] demonstrated that an original cationic polymer 7C1/siRNA complex successfully suppressed Lewis lung carcinoma metastasis. However, such cationic nanoparticle also non-specifically silenced off-target organs.[10,11] In an attempt to eliminate such non-specific silencing, we show that an RGD-LNP had a high selectivity for the metastasis site.

Additionally, although it is well known that the properties of vessels in experimental primary tumors (subcutaneous implantation) are angiogenic, malformed, and leaky in an experimental lung metastasis tumor model (intravenous [i.v.] implantation), specifically delivering nanoparticles to such tissue is unclear. Although recent studies have revealed that cancer cells hijack pre-existing vessels in metastatic lesions, a process known as vessel co-option, the properties of such vessels remain unclear despite their clinical significance.[12,13] Although it has been clearly demonstrated that macromolecules with a prolonged circulation time exhibited a significant therapeutic effect, there is no clear, direct evidence to show that the enhanced permeability of the vasculature in the metastatic lung could be attributed to such a therapeutic effect.[14–16]

Received 29 August 2018; accepted 23 October 2018;
https://doi.org/10.1016/j.omto.2018.10.004.

Correspondence: Hideyoshi Harashima, Molecular Design of Pharmaceutics, Faculty of Pharmaceutical Sciences, Hokkaido University, Kita 12, Nishi 6, Kita-ku, Sapporo 060-0812, Japan.
E-mail: harasima@pharm.hokudai.ac.jp

 © 2018 The Author(s).
This is an open access article under the CC BY license (http://creativecommons.org/licenses/by/4.0/).

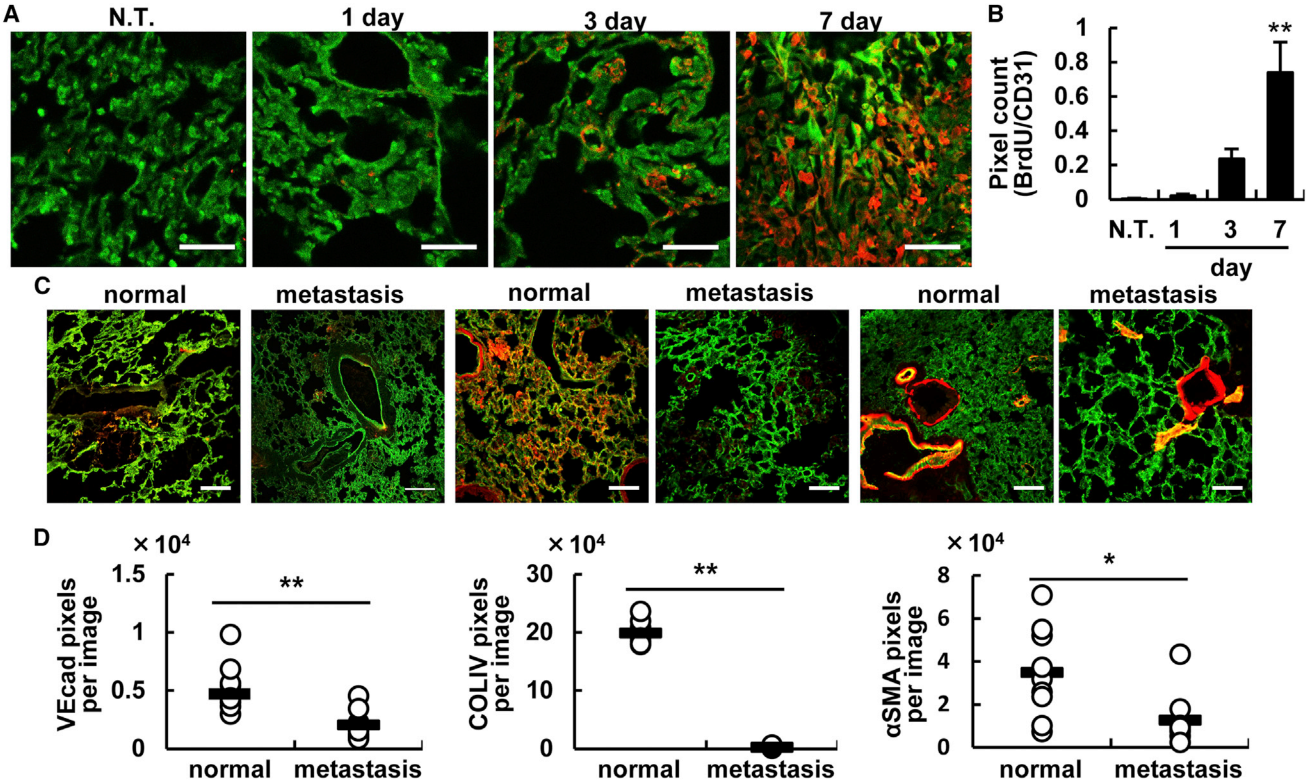

Figure 1. Characterization of the Vasculature in the Lung Metastasis Site

(A) Proliferation of the vasculature at the metastasis site. After BrdU was systemically administered into the tail vein 2 hr before the lungs were excised from the mice that had been inoculated 1, 3, and 7 days before the experiment, the incorporated BrdU was detected by means of an anti-BrdU antibody. Green and red dots indicate vessel and BrdU, respectively. Scale bars, 50 μm. (B) Quantified data of (A) images. The total area of the yellow dots, which denote proliferating endothelial cells, was counted with the ImageJ software. Data represent the mean ± SD. Statistical analyses were performed by ANOVA, followed by the SNK test (n = 6 from 3 independent mice). Statistical analysis was carried out by ANOVA, followed by Bonferroni test. $^{**}p < 0.01$ (versus NT and 1 day). (C) Comparison of the structure of the vasculature in the metastasized lung between normal lung and a lung 14 days after metastasis. The red dots denote VEcad (left), type IV collagen (center), and αSMA (right), respectively. Scale bars, 100 μm. (D) Quantified data of (C) images. Pixels were counted by the ImageJ software in 9 images from 3 independent mice. Statistical analyses were performed by the Student's t test. $^{*}p < 0.05$, $^{**}p < 0.01$.

A recent report revealed that doxorubicin-loading liposomes were not significantly more effective than free doxorubicin in clinical studies.[17] It should therefore be important to confirm whether delivering nanoparticles via leaky tumor vessels (enhanced permeability and retention effect) is effective or not at the metastasis site, since clinical patients suffer, not only from the effects of a primary tumor but also from a metastatic tumor in many cases. In this study, we report the characteristics of vessels in a lung metastasis model, that RGD-LNP would be a useful nanoparticle for the treatment of metastatic cancer, and that nanoparticles with a prolonged circulation time would not tend to accumulate in a metastasized lung.

RESULTS AND DISCUSSION

Permeable Vasculature of Metastatic Breast Cancer

Since the properties of tumor vasculature at a metastasis site and the consequent nanoparticle accumulation have not yet been unveiled,[12,18] we first characterized the tumor vasculature at a metastasis site, namely, breast cancer that had metastasized to the lung. After systemically administering 4T1 cells to mice via the tail vein, the lung was observed by confocal laser-scanning microscopy (CLSM). The numbers of proliferating endothelial cells increased significantly with passing time (Figures 1A and 1B). The cancer cells first became clogged in the vessels and then formed a metastatic region (Figure S1). The endothelial cell adhesion protein vascular endothelial cadherin (VEcad) and type IV collagen (COLIV) containing the basement membrane of the vasculature were significantly reduced in the metastasis lung (Figures 1C and 1D). The alpha smooth muscle actin (αSMA)-positive cells that line the vasculature (pericytes) are considered to be a marker of mature vessels.[19–21] In the metastasis lung, αSMA levels were also significantly decreased (Figure 1E). These results suggest that the vascular permeability in the metastasis lung would be increased and, therefore, had the same characteristic structure as that in the primary tumor. In other words, nanoparticles with a prolonged circulation time could accumulate in tumor tissue via the enhanced permeability and retention (EPR) effect, based on conventional understanding. Further studies directed at the structure

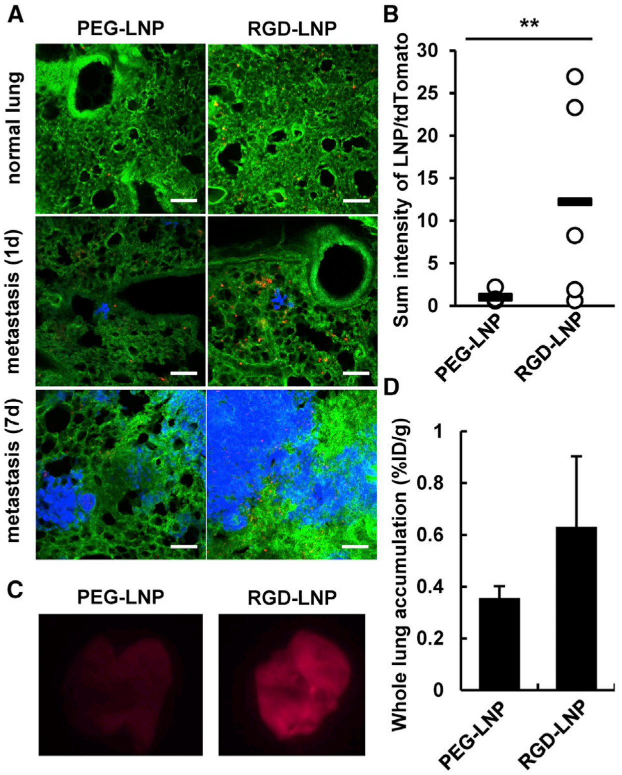

Figure 2. Accumulation of PEG-LNP and RGD-LNP in the Metastasis Lung

(A) Localization of each PEG-LNP or RGD-LNP in the lungs from normal and metastasized mice was observed 7 and 1 and 7 days after the injection of 4T1 cells. Lungs were collected at 24 hr after the injection of each of the LNPs. Blue, green, and red dots indicate 4T1/tdTomato, vessel (Alexa488-labeled antibody), and LNPs (1,1'-dioctadecyl-3,3,3',3'- tetramethylindodicarbocyanine, 4-chlorobenzenesulfonate salt [DiD]) in pseudo-color images, respectively. Scale bars, 100 μm. (B) The obtained images were analyzed using the ImageJ software. Statistical analysis was performed with the Student's t test. $^{**}p < 0.01$. (C) LNP in the metastasis lung (7 days) was observed by FluorVivo. In the images, a red color indicates an LNP. (D) Accumulation of radioisotopically labeled LNP in whole lung. The extent of accumulation per gram of whole lung at 24 hr after the injection was determined by liquid scintillation counting. The percentage of injected dose (%ID) was calculated by comparing radioactivity of the lungs with that of LNP samples. In these experiments, 3 mice were used in independent experiments. Data represent the mean ± SD.

of vessels in a metastasis site with other metastasis models, such as spontaneous and other cancer types, will be needed if progress is to be made regarding nanoparticle-based therapy.

Comparison of PEGylated LNP and RGD-LNP in the Metastasis Lung

The accumulation of polyethylene glycol (PEG)ylated LNP (PEG-LNP) and RGD-LNP in the lung was compared by CLSM 1, at 3 and 7 days after implantation. PEG-LNP levels in the lung tissue were negligible. On the other hand, a larger amount of the RGD-LNP was delivered to the lung vasculature (Figure 2A). The accumulation of RGD-LNP per cancer cell was much higher than that for the PEG-LNP (Figure 2B). An overall observation of lung also showed that RGD-LNP was superior to PEG-LNP in terms of the amount that had accumulated (Figure 2C). When radioisotopically (RI)-labeled LNPs were systemically administered to tumor-bearing mice via the tail vein, the accumulation of RGD-LNP was found to be 1.9-fold higher than that for PEG-LNP at day 7 (Figure 2D). The amount of PEG-LNP that accumulated increased slightly with time, suggesting that vascular permeability contributed slightly to the EPR effect-based delivery of the PEG-LNP in the metastasized lung. However, the effect of vascular permeability on the accumulation of the PEG-LNP appeared to be marginal. Further, the fact that accumulation was independent of the number of post-implantation days on the accumulation of the particles in the liver suggest that this slight increase in the accumulation of PEG-LNP can, in part, be attributed to vascular permeability in the metastasis lung (Figure S2). At the same time, this result suggests that the vascular permeability is a minor factor in the accumulation of nanoparticles in the metastasis lung, at least in the case of this particular experimental lung metastasis model.

Maeda[18] previously reported that Evans Blue, a dye that binds to albumin and is then delivered to a primary tumor via the EPR effect,[22,23] was also delivered to a lung metastasis site. Although we also observed the accumulation of Evans Blue dye at the metastasis site (Figure S3A), the amount of Evans Blue in the whole lung did not appear to be increased significantly at 14 days post-implantation (Figure S3B). This result can be interpreted to mean that the contribution of vasculature permeability in the primary tumor was not substantial, as was observed in the primary tumor subcutaneous model. After a mouse was inoculated both subcutaneously (primary tumor) and systemically (lung metastasis) at the same time that the PEG-LNP was intravenously administered, an enormous amount of PEG-LNP was detected in the primary tumor (Figures 3A and 3B). On the other hand, only small amounts of PEG-LNP were observed in the metastasis lung (Figure 3C). These results suggest that tumor vasculature targeting (RGD-LNP) was a more potent strategy for therapeutic delivery than PEG-LNP. However, the accumulation of RGD-LNP at the metastatic site appeared to be lower than that in the primary tumor.[6]

In previous studies, it was reported that the protein corona absorbed on the surface of a nanoparticle disturbed the capacity for targeting.[24,25] For more efficient targeting, the properties of nanoparticles should be strictly optimized in terms of size, type of ligand used, and related issues. In addition, it also appears that EPR effect-based delivery in lung metastasis is not as effective as that in subcutaneous xenografts. This might provide an explanation for the critical difference in the therapeutic effect of doxorubicin-loaded nanoparticles between clinical and non-clinical conditions.

Knockdown of the Vasculature Gene by RGD-LNP

To evaluate the gene-silencing ability of RGD-LNP in the lung metastasis vasculature, siVEGFR2 was encapsulated in RGD-LNP, and the resulting product was injected into 4T1-bearing mice five times, at 1, 3, 5, 7, and 9 days post-implantation. The lung was

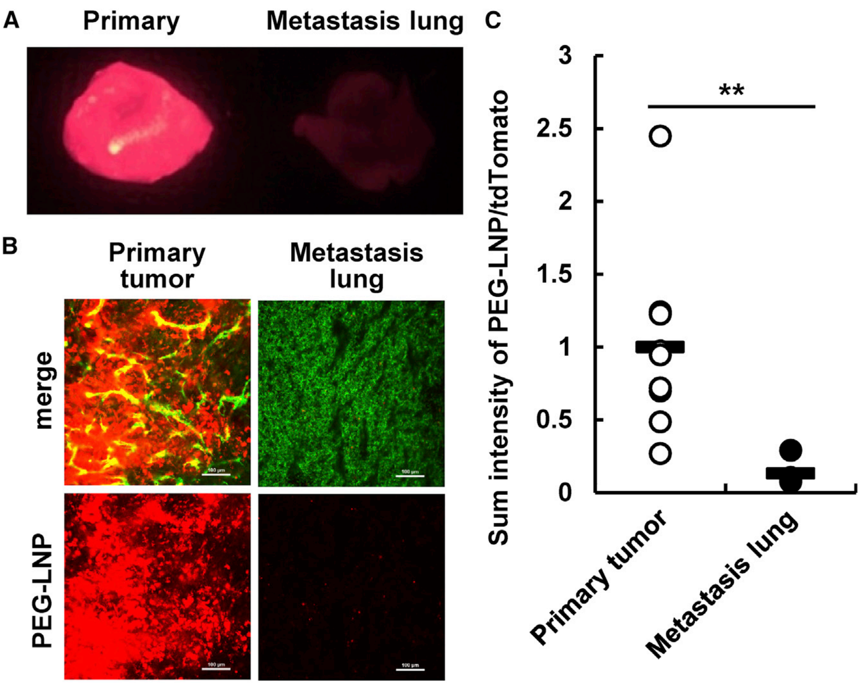

Figure 3. The Accumulation of PEGylated LNP in the Metastasis Lung

(A) Whole fluorescent images of primary tumor and metastasis lungs were taken by FluorVivo 24 hr after the injection of LNPs. Red signals indicate DiD-labeled LNPs. (B) The localization of LNPs in the primary tumor and metastasis lung was observed by CLSM. Green and red signals indicate vessels (Alexa488-labeled antibody) and LNPs (DiD), respectively. At the same time, tdTomato-derived signal was detected, but the tdTomato fluorescence is not indicated in the figures for clarity. (C) Images of (B) were quantified using the ImageJ software. y axis was calculating by dividing the total fluorescent intensity by the tdTomato area derived from 4T1 cell to normalize the amount of LNPs that had accumulated per cancer cell. Statistical analyses were performed by the Student's t test. $^{**}p < 0.01$ (n = 9). In all experiments, 3 mice were used in independent experiments.

immunostained 24 hr after the final injection. VEGFR2 expression was significantly reduced in whole-lung tissue (Figures 4A and 4B). Since VEGFR2 expression was detected both in cancer cells and TECs, its expression in TECs, shown as yellow pixels in Figure 4, was evaluated. Since previous reports showed that VEGFR2 was expressed not only in TECs but also in cancer cells themselves for autocrine proliferative effects, the localization of VEGFR2 was consistent with other general results.[26,27] Further, the cancerous regions and the non-cancerous regions were clearly separated from the whole collected lung tissue. The RGD-LNP injection led to a significant *Vegfr2* mRNA knockdown only in the cancerous region and not in the non-cancer part (Figure 4C). The purity of the cancerous and non-cancerous regions was confirmed by tdTomato mRNA expression. The separated tissues were subjected to nested PCR with the tdTomato-specific primer, which is only expressed in tdTomato/luc2-4T1 cells. The PCR amplicon was detected exclusively in the cancerous region (Figure S4). Considering the above findings, it is clear that the RGD-LNP exerted highly selective gene silencing at the metastasis site.

Therapeutic Effect by RGD-LNP

Lastly, we assessed the therapeutic effect of RGD-LNP against lung metastasis. In this experiment, taking into account the fact that metastatic tumors in clinical patients were already resistant to chemotherapy, we used doxorubicin (DOX)-resistant tdTomato/luc2-4T1 cells, whose sensitivity against DOX was approximately 100-fold lower than that of normal 4T1 cells (Figure S5A). This resistance was canceled by Verapamil, a known P-glycoprotein (Figure S5B).[28] Although we previously reported that siVEGFR2 encapsulated in RGD-LNP could inhibit tumor growth in primary renal cell tumors,[6] the survival for the lung metastasis model was not prolonged (Figure S6). This result might indicate that the supply of oxygen, nutrients, and growth factors needed by the metastatic tumor depends not on angiogenesis but vascular co-option, which mean cancer cells invaded the pre-existing vessels of the metastasized host organ.[12]

We then used siRNA against the delta-like ligand (DLL) 4, which is a well-known endothelial gene that ultimately exerts an inhibitory effect on tumor growth since the inhibition of DLL4 resulted in non-productive angiogenesis.[29,30] This inhibitory effect was reported to be caused by a chaotic vascular network.[31,32] When RGD-LNP encapsulating anti-DLL4 siRNA (siDll4) was injected 8 times at a dose of 2.0 mg/kg, the overall survival of the lung metastasis mouse model was moderately prolonged (Figure 5), but, unfortunately, the results were not statistically significant ($p = 0.0508$, non-treatment [NT] versus RGD-LNP) because of the small sample number. On the other hand, the PEG-LNP (not RGD-modified LNP) and DOX-loaded liposomes exhibited no therapeutic effect. Additionally, when *Dll4* was suppressed in an *in vitro* study with 4T1 cells, no difference on the viability between siDll4 and siRNA against human polo-like kinase 1 (siControl) (Figure S7) was found.

Although DLL4 expression might be also decreased in cancer cells (Figures 4A and 4B), these results suggest that this therapeutic effect can be attributed to a decrease of *Dll4* expression in TECs caused by the RGD-LNP. In addition, in the case of DOX-sensitive 4T1 cells, not only RGD-LNP but also DOX-loaded liposomes showed a therapeutic effect (Figure S8), indicating that the therapeutic effect in the experimental lung metastasis model did not reflect the amount of nanoparticles loaded with the anti-cancer drug that had accumulated. Concerning the primary tumor site, it was reported that DLL4 inhibition caused by the antibody induced a significant delay in primary

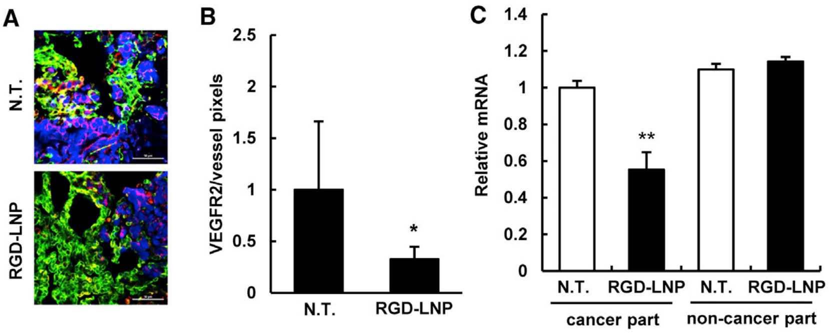

Figure 4. Gene Silencing of the Metastasis Lung by RGD-LNP

(A) VEGFR2 expression was detected by immunostaining. Blue, green, and red colors indicate 4T1/tdTomato, vessels (Alexa488-labeled antibody) and VEGFR2 (Alexa647-labeled antibody) protein, respectively. Scale bars, 50 μm. (B) CLSM images were quantified using the ImageJ software. VEGFR2 expression on vessels, as indicated by yellow dots in the pseudo-images, was quantified. Statistical analyses were performed using the Student's t test. $^{*}p < 0.05$. Data represent the mean ± SD. (C) *Vegfr2* mRNA expression was measured by qRT-PCR after dividing the lung into a cancer region and non-cancer region when RGD-LNPs were administered 3 times each day. These regions were visually separated by collecting the nodules from the whole lung. After isolating total RNA from each collected tissue, VEGFR2 mRNA expression was quantified by qRT-PCR. Statistical analysis was performed by ANOVA, followed by the SNK test. $^{**}p < 0.01$ (n = 4). Data represent the mean ± SD. In all experiments, 4 mice were used in independent experiments.

tumor growth.[33,34] Accordingly, a therapeutic effect against a primary tumor with RGD-LNP would be expected because of our previous report on a gene-silencing effect in primary tumors with RGD-LNP.[6] Further study will be required before anti-cancer drug-loaded nanoparticles can be used with an experimental lung metastasis model.

MATERIALS AND METHODS

General

Distearoyl-*sn*-glycerophosphocholine (DSPC), PEG-distearoyl-*sn*-glycerolphosphoethanolamine (DSPE), PEG-dimyristoyl-glycerol (DMG), and PEG-distearoyl-glycerol (DSG) were purchased from NOF (Tokyo, Japan). YSK05 was synthesized as previously reported.[35] Cyclic RGD (cRGD) was synthesized by Peptides International (Louisville, KY, USA). RPMI-1640, cholesterol (chol), and Hoechst33342 were purchased from Sigma-Aldrich (St. Louis, MO, USA). siRNAs were synthesized by Hokkaido (siVEGFR2: sense, cAA ccA GAG Acc cuc Guu udTsdT; anti-sense, AAA CGA GGG UCU CUG GUU GdTsdT; siDLL4: sense, ucc uGu AuG GGA cAu cuu udTsdT; anti-sense, AAA GAU GUC CcA uAc AGG AdTsdT; lowercase denotes 2′-OMe-modified RNA, "s" denotes phosphorothioate, and "d" denotes DNA). These sequences were previously reported.[10] PBS without Ca^{2+} and Mg^{2+} (PBS [−]) was purchased from Wako Pure Chemical Industries (Osaka, Japan).

Animal Model

BALB/c (4-week-old, female) mice were purchased from SLC Japan (Shizuoka, Japan). The murine breast cancer 4T1 cells expressing fluorescent protein TdTomato and luciferase (4T1-TdTomato/luc2) were systemically administered via the tail vein at a density of 1.0×10^5 cells. To establish DOX-resistant 4T1 cells, 4T1 cells were incubated in RPMI containing 800 nM DOX for over 4 weeks, and the cells were then subjected to single-cell cloning. For maintenance, the obtained DOX-resistant cells were cultured in RPMI containing 800 nM DOX. All animal experimental procedures were carried out in accordance with the Guide for the Care and Use of Laboratory Animals as stated by the NIH, and they were approved by the Hokkaido University Animal Committee.

Preparation of LNPs

First, 2,100 nmol YSK05, 900 nmol chol, and 90 nmol PEG-DMG were dissolved in 400 μL *t*-BuOH. PEG-DMG was altered with PEG-DSG to form PEG-LNP, which was delivered to the tumor site via the EPR effect. In the next step, 200 μL siRNA solution (40–160 μg siRNA in 2 mM citrate buffer [pH 4.0]) was added to the lipid mixture under vigorous mixing. The siRNA and lipid mixture was next diluted with 2.0 mL citrate buffer, and the solution was then rapidly diluted with 4.0 mL PBS (−). The resulting preparation was subjected to twice ultrafiltration with an Amicon Ultra-15 against PBS (−) to remove un-encapsulated siRNA and to reduce the concentration of *t*-BuOH. The solution was mixed with 18 nmol RGD-PEG-DSPE (3 mol% of total lipids) in 7.5% EtOH (v/v, 20 mM citrate buffer [pH 5.5]) for 30 min at 60°C. RGD modification was not carried out in the case of the PEG-LNP. The siRNA recovery ratio and encapsulation efficiency of each of the LNPs were determined by means of a RiboGreen assay, as previously reported.[36] LNPs were characterized using a Zetasizer Nano ZS ZEN3600 (Malvern Instruments, Worchestershire, UK). To labeled LNPs with fluorescence, 0.5 mol% DiD was added to the first lipid mixture.

Analysis of Tumor Vasculature Structure by Immunofluorescence Microscopy

Lung tissues were collected from mice that had been sacrificed under a CO_2 atmosphere. They were filled with PBS (−):optimal cutting temperature (OCT) compound (1:1), and the resulting lung tissues were embedded in OCT compound. The resulting tissues were sliced at a thickness of 5 μm. After placing the slices on MAS-coated glass slides (Matsunami, Tokyo, Japan), they were then incubated with the first antibody solution using a Cy3-labeled anti-αSMA antibody (Sigma-Aldrich, C6198), anti-mouse CD31 Armenian Hamster immunoglobulin G (IgG) (MA31505, Thermo Fisher Scientific), anti-mouse VEcad rat IgG (138001, BioLegend), and anti-bromodeoxyuridine (BrdU) mouse IgG (364101, BioLegend). The slices were then washed twice with PBS (−) and immersed in a second antibody solution containing DyLight488-labeled anti-Armenian hamster IgG goat IgG (405503, BioLegend) for CD31 and AlexaFluor647-labeled anti-Rat IgG goat IgG (A21247, Thermo Fisher Scientific) for 30 min. The slices were covered with a coverslip (Matsunami, 1s) after washing with

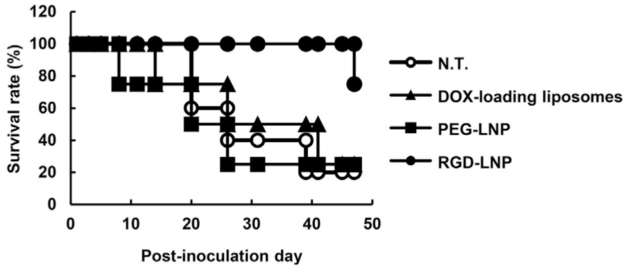

Figure 5. Overall Survival of Metastasized Mice

Mice inoculated with DOX-resistant 4T1 cells were treated with Doxil, PEG-multifunctional envelop-type nano device (MEND) (no RGD modification), and RGD-MEND at 2.0 mg/kg (siRNA) or 3.0 mg/kg (doxorubicin) at days 1, 3, 5, 8, 11, 14, and 20 (n = 4–5).

PBS (−). The tumor slices were observed with a Nikon A1R system (Nikon, Tokyo, Japan) using CFI Plan Apo Lambda 20× or CFI Plan Apo VC 60× water immersion objective lens.

Detection of Proliferating Endothelial Cells in Metastasized Lungs

Mice were intraperitoneally treated with 150 μL 10 mg/mL BrdU solution 2 hr before the metastasized lung was resected. The resected lung was embedded in OCT compound (Sakura Finetek Japan, Tokyo, Japan), and 5-μm-thick slices were produced with a CM3050S (Leica Microsystems, Nussloch, German). For denaturing genomic DNA, the lung sections were treated with 1N HCl for 10 min and then with 2N HCl for 10 min at room temperature. After removing the 2N HCl, the sections were immersed in 0.1 M borate buffer for 12 min. The slice was then washed 3 times with 1% 1% Triton X-100 in 0.1 M PBS (pH 7.4) for 5 min each. After allowing the slice to stand in 1.5% Triton X-100 in 0.1 M PBS (pH 7.4) for 1 hr, it was incubated in anti-BrdU rat IgG (BioLegend) overnight at 4°C, and then a Goat anti-Rat IgG (H+L) Secondary Antibody (Thermo Fisher Scientific) for 30 min.

Observation of the Accumulation of Evans Blue Dye

To observe Evans Blue dye in the metastasis lung, 100 μL 5% solution of Evans Blue was systemically administered to normal or metastasized mice. The lungs were then excised and observed or minced with scissors in 200 μL PBS (−) 30 min after the injection. The extracted Evans Blue was then measured.

Observation of the Accumulation of LNPs

Fluorescent LNPs were systemically administered via the tail vein, and the lungs were excised 24 hrs after the injection of LNPs. The excised lungs were incubated in 10 μg/mL solution of AlexaFluor488-labeled anti-mouse CD31 rat IgG (BioLegend, 102514) and 10 μg/mL Hoechst33342 solution to stain the vessels and nuclei. The whole tissues were mounted on coverslips (Matsunami, 1s) and observed with a Nikon A1R system. The whole image of a lung treated with LNPs was observed using a FluorVivo Imaging System (Indec BioSystem, Santa Clara, CA, USA).

Quantification of mRNA Expression

Using scissors, the cancerous and non-cancerous regions of the lungs were visually isolated and collected 24 hr after the injection of the LNPs. The tissues were then homogenized in TRI Reagent with a PreCelly24 (Bertin Technologies, Montigny-le-Bretonneux, France). The total extracted RNA was then subjected to reverse transcription with a High-Capacity RNA-to-cDNA kit (Thermo Fisher Scientific), according to the manufacturer's instructions. The obtained cDNA was diluted to an appropriate concentration (typically 50-fold), and qPCR was carried out using the diluted cDNA. To clarify whether cancerous regions could be distinguished from non-cancerous regions of lungs, nested PCR was performed.

Quantification of the Accumulation of LNPs by Radioisotope

To label LNPs with a radioisotope, cholesteryl hexadecyl ether (CHE) was added to the lipid composition at the preparation step. Lungs were collected 24 hr after the LNPs containing approximately 1,000,000 dpm had been intravenously administered via the tail vein. The excised lungs were minced with scissors and then dissolved in Soluene-350 (PerkinElmer, Boston, MA) at 50°C overnight. A 20 mL portion of HionicFluor (PerkinElmer) was added to the dissolved solution, and the radioactivity was then measured using an LSC-6100 counter (ALOKA, Tokyo, Japan).

Statistical Analysis

The Student's t test was carried out for pairwise comparison. For comparison among three or more groups, the non-repeated ANOVA (nrANOVA), followed by Bonferroni test or Student-Newman-Keuls (SNK) test, was used. p value < 0.05 was regarded as being a statistically significant difference.

SUPPLEMENTAL INFORMATION

Supplemental Information includes eight figures and can be found with this article online at https://doi.org/10.1016/j.omto.2018.10.004.

AUTHOR CONTRIBUTIONS

Y.S. and H.H. designed the whole study. T.H. was involved in almost all of the experiments. A.K. and W.M. performed *in vivo* experiments. Y.H. carried out the *in vitro* competitive inhibition assay.

CONFLICTS OF INTEREST

The authors have no conflicts of interest directly relevant to the content of this article.

ACKNOWLEDGMENTS

The authors wish to thank Dr. Milton S. Feather for appropriately modifying the manuscript. This study was supported, in part, by research grants from Research on the Development of New Drugs, a Health and Labour Sciences Research Grant, JSPS KAKENHI grant JP18K18351, and The Mochida Memorial Foundation for Medical and Pharmaceutical Research.

REFERENCES

1. Hanahan, D., and Weinberg, R.A. (2000). The hallmarks of cancer. Cell *100*, 57–70.

2. Sporn, M.B. (1996). The war on cancer. Lancet *347*, 1377–1381.
3. Steeg, P.S. (2016). Targeting metastasis. Nat. Rev. Cancer *16*, 201–218.
4. Naresh, K.N., Nerurkar, A.Y., and Borges, A.M. (2001). Angiogenesis is redundant for tumour growth in lymph node metastases. Histopathology *38*, 466–470.
5. Sakurai, Y., Hatakeyama, H., Sato, Y., Hyodo, M., Akita, H., and Harashima, H. (2013). Gene silencing via RNAi and siRNA quantification in tumor tissue using MEND, a liposomal siRNA delivery system. Mol. Ther. *21*, 1195–1203.
6. Sakurai, Y., Hatakeyama, H., Sato, Y., Hyodo, M., Akita, H., Ohga, N., Hida, K., and Harashima, H. (2014). RNAi-mediated gene knockdown and anti-angiogenic therapy of RCCs using a cyclic RGD-modified liposomal-siRNA system. J. Control. Release *173*, 110–118.
7. Hada, T., Sakurai, Y., and Harashima, H. (2015). Optimization of a siRNA Carrier Modified with a pH-Sensitive Cationic Lipid and a Cyclic RGD Peptide for Efficiently Targeting Tumor Endothelial Cells. Pharmaceutics *7*, 320–333.
8. Thurston, G., McLean, J.W., Rizen, M., Baluk, P., Haskell, A., Murphy, T.J., Hanahan, D., and McDonald, D.M. (1998). Cationic liposomes target angiogenic endothelial cells in tumors and chronic inflammation in mice. J. Clin. Invest. *101*, 1401–1413.
9. Santel, A., Aleku, M., Röder, N., Möpert, K., Durieux, B., Janke, O., Keil, O., Endruschat, J., Dames, S., Lange, C., et al. (2010). Atu027 prevents pulmonary metastasis in experimental and spontaneous mouse metastasis models. Clin. Cancer Res. *16*, 5469–5480.
10. Dahlman, J.E., Barnes, C., Khan, O., Thiriot, A., Jhunjunwala, S., Shaw, T.E., Xing, Y., Sager, H.B., Sahay, G., Speciner, L., et al. (2014). In vivo endothelial siRNA delivery using polymeric nanoparticles with low molecular weight. Nat. Nanotechnol. *9*, 648–655.
11. Aleku, M., Schulz, P., Keil, O., Santel, A., Schaeper, U., Dieckhoff, B., Janke, O., Endruschat, J., Durieux, B., Röder, N., et al. (2008). Atu027, a liposomal small interfering RNA formulation targeting protein kinase N3, inhibits cancer progression. Cancer Res. *68*, 9788–9798.
12. Donnem, T., Hu, J., Ferguson, M., Adighibe, O., Snell, C., Harris, A.L., Gatter, K.C., and Pezzella, F. (2013). Vessel co-option in primary human tumors and metastases: an obstacle to effective anti-angiogenic treatment? Cancer Med. *2*, 427–436.
13. Holash, J., Maisonpierre, P.C., Compton, D., Boland, P., Alexander, C.R., Zagzag, D., Yancopoulos, G.D., and Wiegand, S.J. (1999). Vessel cooption, regression, and growth in tumors mediated by angiopoietins and VEGF. Science *284*, 1994–1998.
14. Zhang, Z., Niu, B., Chen, J., He, X., Bao, X., Zhu, J., Yu, H., and Li, Y. (2014). The use of lipid-coated nanodiamond to improve bioavailability and efficacy of sorafenib in resisting metastasis of gastric cancer. Biomaterials *35*, 4565–4572.
15. Parhi, P., Suklabaidya, S., and Kumar Sahoo, S. (2017). Enhanced anti-metastatic and anti-tumorigenic efficacy of Berbamine loaded lipid nanoparticles in vivo. Sci. Rep. *7*, 5806.
16. Yu, F., Li, J., Xie, Y., Sleightholm, R.L., and Oupický, D. (2016). Polymeric chloroquine as an inhibitor of cancer cell migration and experimental lung metastasis. J. Control. Release *244*, 347–356.
17. Petersen, G.H., Alzghari, S.K., Chee, W., Sankari, S.S., and La-Beck, N.M. (2016). Meta-analysis of clinical and preclinical studies comparing the anticancer efficacy of liposomal versus conventional non-liposomal doxorubicin. J. Control. Release *232*, 255–264.
18. Maeda, H. (2015). Toward a full understanding of the EPR effect in primary and metastatic tumors as well as issues related to its heterogeneity. Adv. Drug Deliv. Rev. *91*, 3–6.
19. Fischer, B., Fischer, U., and Lehrl, S. (1981). [Strategy of health education in the healing procedure (II)]. Offentl. Gesundheitswes. *43*, 617–623.
20. Viallard, C., and Larrivée, B. (2017). Tumor angiogenesis and vascular normalization: alternative therapeutic targets. Angiogenesis *20*, 409–426.
21. Hida, K., Maishi, N., Sakurai, Y., Hida, Y., and Harashima, H. (2016). Heterogeneity of tumor endothelial cells and drug delivery. Adv. Drug Deliv. Rev. *99*, 140–147.
22. Kratz, F. (2008). Albumin as a drug carrier: design of prodrugs, drug conjugates and nanoparticles. J. Control. Release *132*, 171–183.
23. Duncan, R., Sat-Klopsch, Y.N., Burger, A.M., Bibby, M.C., Fiebig, H.H., and Sausville, E.A. (2013). Validation of tumour models for use in anticancer nanomedicine evaluation: the EPR effect and cathepsin B-mediated drug release rate. Cancer Chemother. Pharmacol. *72*, 417–427.
24. Xiao, W., Xiong, J., Zhang, S., Xiong, Y., Zhang, H., and Gao, H. (2018). Influence of ligands property and particle size of gold nanoparticles on the protein adsorption and corresponding targeting ability. Int. J. Pharm. *538*, 105–111.
25. Zhang, H., Wu, T., Yu, W., Ruan, S., He, Q., and Gao, H. (2018). Ligand Size and Conformation Affect the Behavior of Nanoparticles Coated with in Vitro and in Vivo Protein Corona. ACS Appl. Mater. Interfaces *10*, 9094–9103.
26. Weigand, M., Hantel, P., Kreienberg, R., and Waltenberger, J. (2005). Autocrine vascular endothelial growth factor signalling in breast cancer. Evidence from cell lines and primary breast cancer cultures in vitro. Angiogenesis *8*, 197–204.
27. Aesoy, R., Sanchez, B.C., Norum, J.H., Lewensohn, R., Viktorsson, K., and Linderholm, B. (2008). An autocrine VEGF/VEGFR2 and p38 signaling loop confers resistance to 4-hydroxytamoxifen in MCF-7 breast cancer cells. Mol. Cancer Res. *6*, 1630–1638.
28. Aller, S.G., Yu, J., Ward, A., Weng, Y., Chittaboina, S., Zhuo, R., Harrell, P.M., Trinh, Y.T., Zhang, Q., Urbatsch, I.L., and Chang, G. (2009). Structure of P-glycoprotein reveals a molecular basis for poly-specific drug binding. Science *323*, 1718–1722.
29. Noguera-Troise, I., Daly, C., Papadopoulos, N.J., Coetzee, S., Boland, P., Gale, N.W., Lin, H.C., Yancopoulos, G.D., and Thurston, G. (2006). Blockade of Dll4 inhibits tumour growth by promoting non-productive angiogenesis. Nature *444*, 1032–1037.
30. Ridgway, J., Zhang, G., Wu, Y., Stawicki, S., Liang, W.C., Chanthery, Y., Kowalski, J., Watts, R.J., Callahan, C., Kasman, I., et al. (2006). Inhibition of Dll4 signalling inhibits tumour growth by deregulating angiogenesis. Nature *444*, 1083–1087.
31. Li, J.L., and Harris, A.L. (2009). Crosstalk of VEGF and Notch pathways in tumour angiogenesis: therapeutic implications. Front. Biosci. *14*, 3094–3110.
32. Yan, M., and Plowman, G.D. (2007). Delta-like 4/Notch signaling and its therapeutic implications. Clin. Cancer Res. *13*, 7243–7246.
33. Liu, S.K., Bham, S.A., Fokas, E., Beech, J., Im, J., Cho, S., Harris, A.L., and Muschel, R.J. (2011). Delta-like ligand 4-notch blockade and tumor radiation response. J. Natl. Cancer Inst. *103*, 1778–1798.
34. Hoey, T., Yen, W.C., Axelrod, F., Basi, J., Donigian, L., Dylla, S., Fitch-Bruhns, M., Lazetic, S., Park, I.K., Sato, A., et al. (2009). DLL4 blockade inhibits tumor growth and reduces tumor-initiating cell frequency. Cell Stem Cell *5*, 168–177.
35. Sato, Y., Hatakeyama, H., Sakurai, Y., Hyodo, M., Akita, H., and Harashima, H. (2012). A pH-sensitive cationic lipid facilitates the delivery of liposomal siRNA and gene silencing activity in vitro and in vivo. J. Control. Release *163*, 267–276.
36. Sakurai, Y., Hada, T., and Harashima, H. (2016). Preparation of a Cyclic RGD: Modified Liposomal SiRNA Formulation for Use in Active Targeting to Tumor and Tumor Endothelial Cells. Methods Mol. Biol. *1364*, 63–69.

OMTO, Volume *11*

Supplemental Information

Effective Therapy Using a Liposomal siRNA that Targets the Tumor Vasculature in a Model Murine Breast Cancer with Lung Metastasis

Yu Sakurai, Tomoya Hada, Akari Kato, Yuta Hagino, Wataru Mizumura, and Hideyoshi Harashima

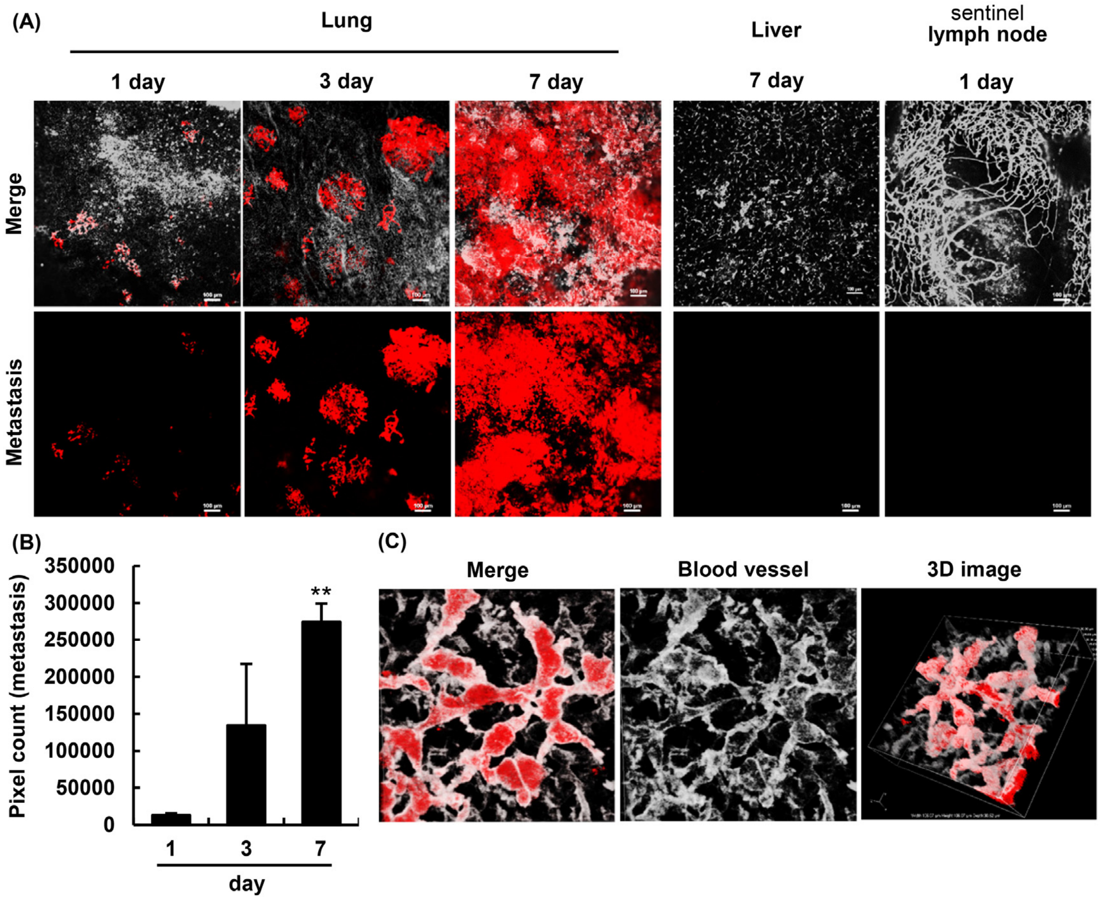

Supplemental Figure 1 Proliferation properties of 4T1-tdTomato/luc cells in the lung. (A) Confocal images of metastasized lung, liver and sentinel lymph node were observed at 1, 3 or 7 days after the inoculation. Red and gray dots indicate 4T1 cells and vessels, respectively. Bars are 100 µm. (B) Red pixels were counted by ImageJ software. Statistical analysis was performed by ANOVA, followed by SNK test. (n=9). **: $p < 0.01$. (C) 3D images of the lung were observed 1 day after the inoculation.

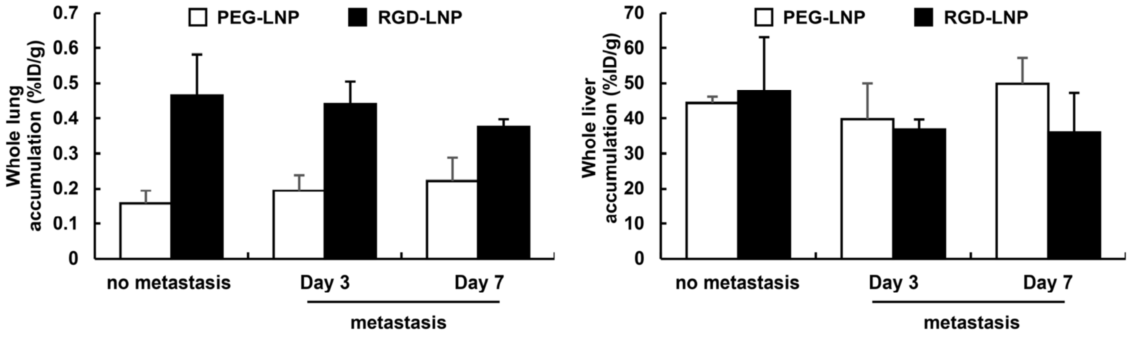

Supplemental Figure 2 PEG- and RGD-LNP accumulation in the lung and liver. Normal mice or metastasized mice were inoculated with radioisotope-labeled LNPs at 3 days or at 7 days. Lung and liver were excised 24 hours after the injection of LNPs, and the radio-activity in the dissolved lung and liver was detected by liquid scintillation counting. Data represent the mean ± SD.

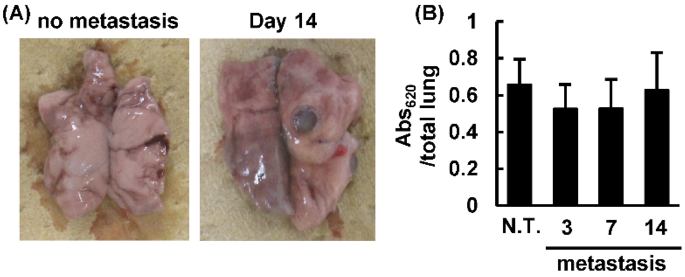

Supplemental Figure 3 Permeability of the metastasized lung vasculature. (A) Visual images of lung samples from mice that were treated with Evans Blue dye. (B) The absorbance of evans blue dye was measured by absorption spectrophotometer at 605 nm after the excised lungs were exhaustively minced. n=3~6. Data represent the mean ± SD.

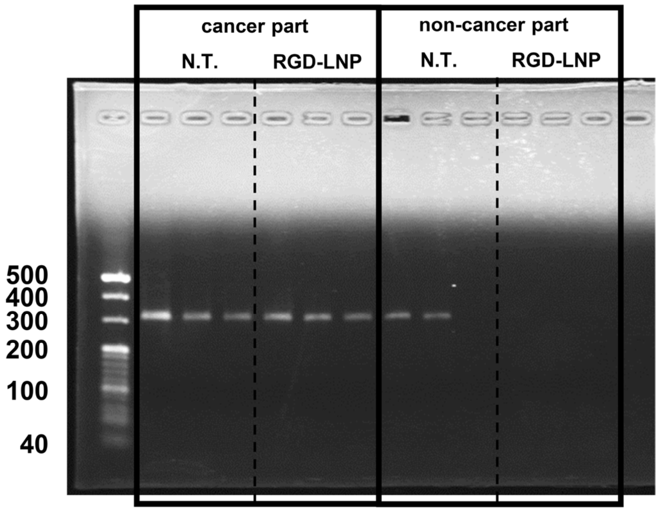

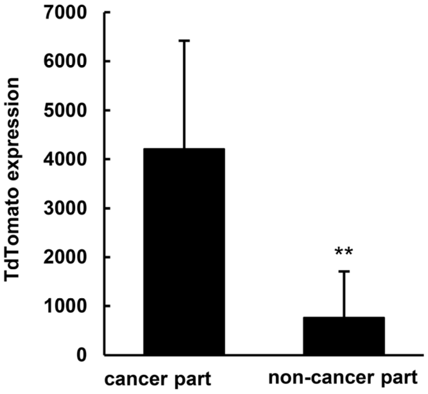

Supplemental Figure 4 Confirmation that the cancerous region was isolated from the non-cancerous region. Cancer and non-cancer regions were isolated based on visual observation, and total RNA was then extracted with TRIzol (Invitrogen). The tdTomato mRNA was then amplified by nested PCR, and the PCR amplicons were then detected by agarose electrophoresis. The electrophoresis images were quantified by ImageJ software. Statistical analyses were performed using the Student's t-test. **: $p < 0.01$ (n=6). Data represent the mean ± SD.

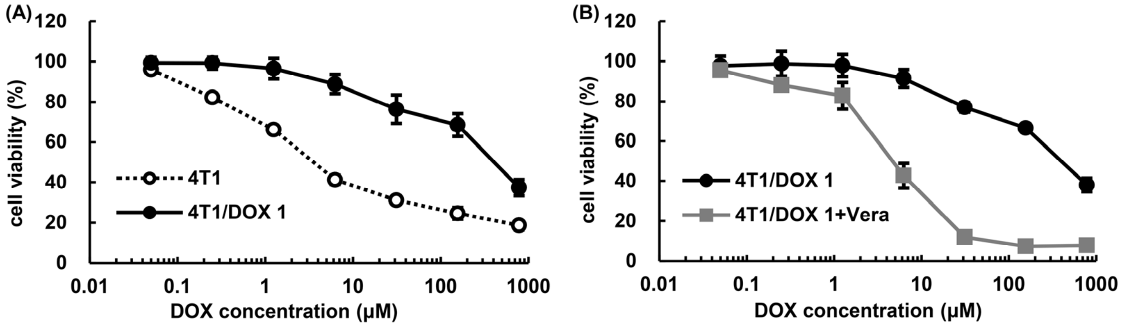

Supplemental Figure 5 Characterization of doxorubicin (DOX)-resistant 4T1 cells (4T1/DOX 1). (A) The sensitivity of 4T1 and 4T1/DOX 1 cells against DOX was measured after treating the cells with 0 – 780 μM DOX. The viability of cells was determined by a WST-8 assay. (B) To elucidate the mechanism for DOX resistance, cells were treated with 0.1 mM Verapamil 1 hr before the addition of DOX (Vera). Data represent the mean ± SD.

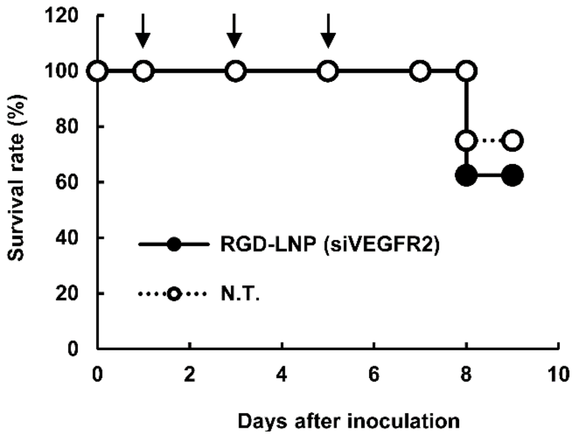

Supplemental Figure 6 The therapeutic effect of RGD-LNP encapsulating siRNA against VEGFR2 (siVEGFR2). RGD-LNP containing siVEGFR2 was administered via the tail vein at a dose of 3.0 mg/kg. Arrows denote the points of injection.

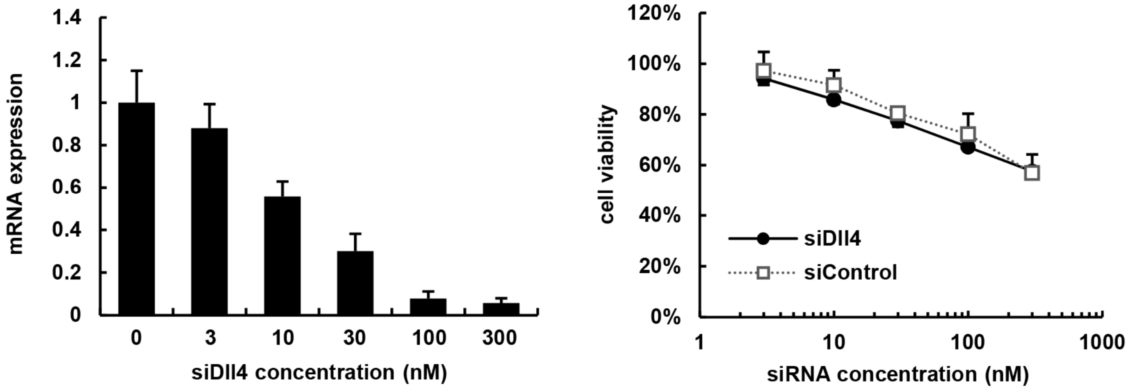

Supplemental Figure 7 Cell killing effect by siDll4. Cells were transfected with siDll4 or siRNA against human polo-like kinase 1 (siControl) by RNAiMAX at the indicated concentrations. Dll4 expression was determined by quantitative RT-PCR. The viability of cells was determined by a WST-8 assay 24 hours after the transfection. Data represent the mean ± SD.

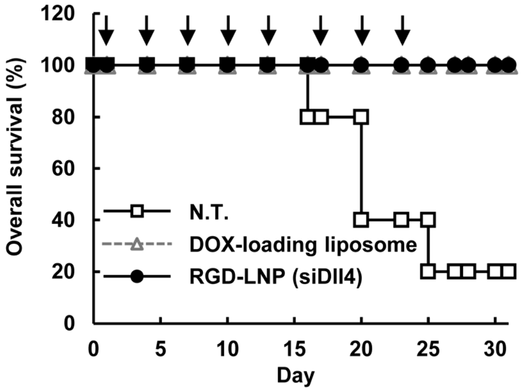

Supplemental Figure 8 The therapeutic effect of RGD-LNP and DOX-loaded liposome in DOX-sensitive 4T1 cells. Mice were treated with RGD-LNP encapsulating siDll4 or DOX-loaded liposome at 3 mg siRNA/kg or 3 mg DOX/kg. Arrows indicate the point of injection of the therapeutics.
